# Serum Retinol Concentrations in Mothers and Newborns at Delivery in a Public Maternity Hospital in Recife, Northeast Brazil

**Published:** 2014-03

**Authors:** Taciana Fernanda dos Santos Fernandes, Luciana Marques Andreto, Carmina Silva dos Santos Vieira, Ilma Kruze Grande de Arruda, Alcides da Silva Diniz

**Affiliations:** Postgraduate Program in Nutrition, Universidade Federal de Pernambuco. Recife, PE, Brazil

**Keywords:** Case series, Mother-infant dyad, Serum retinol, Vitamin A deficiency, Brazil

## Abstract

Serum retinol concentrations were compared in a consecutive series of 65 mother-newborn pairs at delivery in a public maternity hospital in Recife, Brazil, from January to August 2008 and examined their association with biological, socioeconomic, environmental and obstetrical characteristics. Serum retinol concentrations of the newborns (umbilical cord) and mothers (brachial venipuncture) were analyzed by high-performance liquid chromatography. Prevalence of low (<0.70 µmol/L) and inadequate (<1.05 µmol/L) serum retinol concentrations were observed in 23.1% (95% CI 13.9-35.5) and 23.0% (95% CI 13.5-35.8) among newborns and mothers respectively. The serum retinol distribution was lower in male than female infants (-0.4 µmol/L, p=0.01) and, across both sexes, concentrations in paired newborn and mother were correlated (r=0.27, p=0.04). Further, maternal status explains only 7% of the variance in retinol concentrations in newborn's cord plasma. Among mothers delivering in public facilities in Recife, hypovitaminosis may exist.

## INTRODUCTION

Vitamin A is an essential nutrient for cell maintenance and differentiation, the visual cycle (1), child's development and growth (2), and immune response (3). A deficiency of this micronutrient may lead to ocular alterations (xerophthalmia) and increase the risks of infant (4), child (5) and maternal (6) morbidity and mortality.

Vitamin A deficiency (VAD) is considered to be a public-health problem present in more than 70 countries worldwide. Current estimates indicate that approximately 190 million preschool children and 20 million pregnant women have this deficiency. Approximately 7 million of these children have xerophthalmia, and 9-10 million women suffer from night blindness during pregnancy (7). Estimates of this nutritional burden are generally lower for Latin America (7); however, VAD remains a problem among children in areas, such as Northeastern Brazil where it occurs in association with other conditions, such as protein-energy malnutrition and anaemia (8).

In Southeastern Brazil, maternal VAD also exists, occurring clinically as night blindness during pregnancy (9). Its health implications in the region remain unclear, although elsewhere in South Asia, maternal night blindness has been associated with increased risk of infectious diseases, obstetric complications, and mortality among both mothers and infants (10-14), suggesting that both infant and mother share health risks of deficiency. Across Brazil, studies suggest that newborns may be at risk of vitamin A deficiency. At maternity wards in Campinas, São Paulo Rondó *et al.* (15) encountered VAD (serum retinol <0.70 µmol/L) in 14.6% of neonates with weights appropriate for gestational age (AGA). Saunders *et al.* (9) measured serum retinol concentrations in umbilical cord-blood obtained from a university hospital in Rio de Janeiro where he reported 45% of neonates to have inadequacy of vitamin A status (<1.05 µmol/L).

An infant acquires maternal vitamin A in two ways: through the placenta during pregnancy and postnatally via breastmilk. While the placenta plays a crucial role in materno-foetal nutrient transfer mechanisms of regulating transplacental flux of vitamin A in humans, these mechanisms are still poorly understood (16). A highly-specific receptor for the retinol-binding protein (RBP) is expressed in human placental brush border membranes and may regulate retinol uptake through binding of holo-RBP (17), which, in turn, would control the accumulation of retinol in the placenta and its transfer to the foetus as was shown by experiments with human perfused placenta (18). The mechanism may also allow depletion of liver reserves in the neonate, necessitating that the infant builds its liver reserves during the first six months, preferably through breastfeeding. During lactation, the efficiency of transfer of vitamin A to the infant is about 60 times greater than during pregnancy (19). Breastmilk provides 80% of the total vitamin A required by a child in the first two years of life (20).

Few studies have adequately explored the co-occurrence of VAD in the mother-neonate dyad.

The aim of this study was to evaluate the association between serum retinol concentrations in mothers and their newborns and examine these relationships with respect to biological, socioeconomic, environmental and obstetric factors in a typical, lower-middle, urban class of women who gave birth in a public hospital in northern Brazil where 80% of mothers deliver their babies in similar hospital settings.

## MATERIALS AND METHODS

### Design and population of the study

The study consisted of a consecutive series of 65 eligible mothers who gave birth to term infants of both sexes during a daytime on weekdays, from January to August 2008, in the maternity ward of the Instituto de Medicina Integral Professor Fernando Figueira (IMIP). Mothers with high-risk pregnancies were excluded, and 12 eligible mothers refused to participate in the study. IMIP is a state hospital and referral centre for the supervision of high-risk pregnancies. The mean number of births per month in the hospital is 400; records show 42% to be associated with low obstetric risk.

Eligible infants required to be born to women triaged on the maternity ward and classified as being at low obstetric risk, living in the city or metropolitan area of Recife, and who signed an informed consent form to participate. Neonates whose mothers had complications during labour that prevented the collection of blood from the umbilical cord were excluded from the study as were those who were born at night and during weekends when it was not possible to carry out the study procedures.

Following birth, 12.3% of the selected children had Apgar scores of 4 to 8 in the first minute after birth; in the fifth minute after birth, all of the children had an Apgar score of >8.

### Data collection

#### Biochemical data

We collected 5 mL of blood from each of 65 mothers via brachial venipuncture during maternity triage, and 5 mL of blood was collected from the umbilical cords of the neonates in the delivery room. For the retinolaemia analysis, 2 mL of each blood sample was placed in a labelled tube, and the tube was wrapped in aluminium foil to protect it from light. After complete coagulation, the samples were centrifuged at 3,000xg for 10 minutes to achieve total serum separation, and the serum was transferred to Eppendorf tubes and stored at −20 ºC. The serum samples were transported to the Centre for Research on Micronutrients (CIMICRON), Universidade Federal da Paraíba where these were maintained at correct temperature throughout the analysis (-20 ºC). High-performance liquid chromatography (HPLC) was used for determining serum retinol concentrations, employing the technique established by Furr *et al.* (21). Newborns’ serum retinol levels were classified according to World Health Organization (WHO) (22) recommendations: deficient (<0.35 µmol/L), low (0.35-0.70 µmol/L), marginal (0.70-1.05 µmol/L), or adequate (>1.05 µmol/L). Maternal retinol was categorized as inadequate (<1.05 µmol/L) or adequate (>1.05 µmol/L) as no women had low levels of vitamin A (<0.70 µmol/L).

To test for haemoglobinaemia, 3 mL of the collected blood sample was placed in a heparinized vacuum tube and forwarded to the maternity ward laboratory for analysis by electronic cell counter (Sismex SF 3000 Automated Haematology Analyser, GMI, Inc., Ramsey, MN, USA). For a diagnosis of anaemia in women, a cutoff point of <11.0 g/dL haemoglobin (Hb) was used (23). For a diagnosis of neonatal anaemia, a cutoff of <13.5 g/dL Hb was used (24).

#### Anthropometry

Length and weight measurements in infants were done and recorded immediately after birth according to routine procedures. Birthweight was classified as low if it was <2,500 g, insufficient if it was between 2,500 and 2,999 g, and sufficient if ≥3,000 g (25). In addition, length-for-age and body mass index-for-age indices were calculated, expressed as z-scores, and evaluated using Anthro 2005 software (version 2) (WHO, Geneva, CH).

#### Socioeconomic, biological and obstetric data

We employed reported per-capita household income as a socioeconomic status (SES) variable, expressed as a fraction of the Brazilian minimum wage (MW), and categorized into the following three groups: <¼ MW (<US$ 62.2), ¼-½ MW (US$ 62.2-124.4), and ≥½ MW (≥US$ 124.4) where 1 MW=US$ 248.8. A second SES variable was maternal education, categorized as having <8 years and ≥11 years of schooling. In terms of obstetric variables, the number of prenatal visits made by the women during pregnancy was recorded; ≥6 consultations were considered to be adequate, in accordance with the recommendations of the Ministry of Health of Brazil. Maternal parity was categorized into primiparous (study pregnancy was for the first time) or multiparous (study pregnancy was of 2nd or higher order). Maternal age and the condition of home sanitation were recorded, with the latter considered adequate if all three of the following conditions were met: the household water supply was connected to the public network with internal plumbing, sewage was connected to the public network, and refuse (waste material) was regularly collected.

### Data analysis

Database construction was performed using Epi Info (version 6.04) (WHO/CDC, Atlanta, GA, USA), and statistical analysis was performed using the Statistical Package for Social Sciences (SPSS) for Windows (version 12.0) (SPSS Inc., Chicago, IL, USA). Distributions of serum retinol concentrations were tested for normality by the Kolmogorov-Smirnov Test and expressed as mean and standard deviation. Dichotomous distributions were expressed as a proportion with 95% confidence interval derived on binomial assumptions.

Serum retinol distributions of infants were compared by sex, anthropometric status, and socioeconomic characteristics, using the Student's *t*-test and analysis of variance (ANOVA) to compare two or more than two groups respectively. Linear associations between serum retinol concentrations in mother-infant dyads were evaluated using the Pearson's correlation test. A significance level of 5% was required to reject the null hypothesis.

### Ethical aspects

The research protocol was approved by the Ethics Committee of IMIP. All women and infants presenting with anaemia were monitored by the research doctor, were given specific treatment with iron salts, and encouraged to breastfeed exclusively. No drug treatment was provided for women with VAD because they were given a megadose of vitamin A (200,000 IU) in the immediate postpartum period, following the protocol of the Vitamin A Supplementation Programme developed by the Ministry of Health of Brazil.

## RESULTS

Sixty-five neonates born to eligible, consenting mothers were studied. Characteristics of mother-infant dyads are described in Table 1. Among the infants, 56.9% were female, and 72.3% were born with a weight >3,000 g; however, when the body mass index-for-age (>1 z-score) was used, 18% of all newborns were classified as overweight. When the length-for-age index was evaluated, 8.2% of the neonates had linear growth retardation (<-2 z-score).

Regarding maternal characteristics, 84.6% of the women were 20 years of age or older, 78.4% had more than eight years of formal schooling, 87.0% lived in locations with adequate sanitation, and 76.5% had a per-capita income lower than half the minimum wage.

Among the neonates, 23.1% had low retinol concentrations (VAD), and 43.1% were anaemic. Twenty-three percent of mothers had inadequate levels of serum retinol, and 17.5% had anaemia (Table 2).

The overall mean±SD of serum retinol among neonates was 1.13±0.60 µmol/L (Table 3). When the newborn serum retinol distribution was stratified across risk factors, we observed that male infants had a lower mean value than female infants (-0.4 µmol/L, p=0.01), a difference that did not remain statistically significant after adjusting for birthweight and length (data not shown). No other differences in serum retinol were observed across neonatal strata of birthweight, body mass index-for-age and length-for-age. The overall mean±SD of maternal serum retinol concentration was 1.46±0.63 µmol/L. No associations were observed between maternal serum retinol and neonatal anthropometric status (Table 3).

**Table 1.** Characteristics of mother-infant dyads at IMIP, 2008

Characteristics	n	%	95% CI
Neonatal			
Sex			
Male	28	43.1	31.1-55.9
Female	37	56.9	44.1-68.9
Total	65	100.0	
Birthweight (g)			
>2,500-3,000	18	27.7	17.6-40.4
>3,000	47	72.3	59.6-82.3
Total	65	100.0	
Length-for-age z-score			
< −2	05	8.2	3.1-18.8
≥ −2	56	91.8	81.2-96.9
Total	61	100.0	
Body mass index-for-age (z-score)			
≥ −2 to ≤ +1	50	82.0	69.1-90.2
> +1 to ≤ +2	11	18.0	9.4-30.0
Total	61	100.0	
Maternal			
Schooling (years)			
<8	14	21.5	12.7-33.8
8-11	16	24.6	15.1-37.1
≥11	35	53.8	41.1-66.1
Total	65	100.0	
Per-capita income (MWs)			
<¼ MW	18	28.1	17.9-40.9
¼ - ½ MW	31	48.4	35.9-61.2
≥½ MW	15	23.4	14.1-36.0
Total	64	100.0	
Age (years)			
<20	10	15.4	8.0-26.9
≥20	55	84.6	73.1-92.0
Total	65	100.0	
Sanitation			
Adequate	47	87.0	60.7-83.8
Inadequate	07	13.0	4.6-20.8
Total	61	100.0	
Prenatal consultations (number)			
<6	22	33.8	22.9-46.7
≥6	43	66.2	53.5-77.1
Total	65	100.0	

**Table 2.** Concentrations of serum retinol and haemoglobin in the mother-infant dyads at IMIP, 2008

Biochemical variable	n	%	95% CI
Neonatal			
Serum retinol (µmol/L)			
<0.70	15	23.1	13.9-35.5
0.70-1.05	21	32.3	21.5-45.2
≥1.05	29	44.6	32.5-57.4
Total	65	100.0	
Haemoglobin (g/dL)			
<13.6	25	43.1	27.3-53.4
≥13.6	33	56.9	43.3-69.6
Total	58	100.0	
Maternal			
Serum retinol (µmol/L)			
<1.05	14	23.0	13.5-35.8
≥1.05	47	77.0	64.1-86.5
Total	61	100.0	
Haemoglobin (g/dL)			
<11	11	17.5	9.4-29.5
≥11	52	82.5	70.5-90.6
Total	63	100.0	

**Table 3.** Neonatal and maternal serum retinol concentration by sex and anthropometric status

Characteristics	Serum retinol (µmol/L)							
	Neonatal				Maternal			
	n	Mean	SD	p*	n	Mean	SD	p*
All infants	65	1.13	0.60		61	1.46	0.63	
Sex								
Male	28	0.91	0.43	0.01	27	1.35	0.46	0.22
Female	37	1.29	0.67		34	1.56	0.73	
Birthweight (g)								
<3,000	18	1.18	0.85	0.66	17	1.65	0.65	0.17
≥3,000	47	1.11	0.49		44	1.40	0.62	
Body mass index-for-age (z-score)								
≥ −2 to ≤ +1	50	1.13	0.85	0.78†	47	1.49	0.66	0.65†
> +1 to ≤ +2	09	1.26	0.68		08	1.38	0.60	
> +2 to ≤ +3	02	0.96	0.44		02	1.87	0.30	
Length-for-age (z-score)								
≥ −3 to < −2	05	1.09	0.52	0.83	05	1.26	0.67	0.41
≥ −2	56	1.15	0.62		52	1.51	0.65	

*Student's *t*-test for unpaired data; †ANOVA

There were two noteworthy biochemical associations: the neonatal serum retinol concentration was positively correlated with maternal serum retinol concentration (r=0.27, p=0.04, n=61), indicating that the latter explained 7% of the variance in this measure of infant's status; secondly, within neonates, a negative correlation existed between neonatal serum retinol and blood haemoglobin concentrations (r=- 0.26, p=0.05, n=65).

No associations were observed between neonatal serum retinol and household socioeconomic status or adequacy of sanitation, the number of prenatal consultations, or maternal parity or age (data not shown).

## DISCUSSION

A prevalence of ~23% for inadequacy and deficiency in serum retinol concentrations among mothers and newborns respectively suggests that vitamin A deficiency is a problem among women at low obstetric risk, who present for delivery at public hospitals, such as the Instituto de Medicina Integral Professor Fernando Figueira in Northeastern Brazil.

Saunders *et al*. (9) reported results similar to those of this study when they conducted a study of mothers and their infants in a university hospital in Rio de Janeiro. The authors noted that inadequate serum concentrations of retinol (<1.05 µmol/L) were observed in 24% of mothers and 45% of newborns, based on umbilical cord-blood samples. Among neonates born at maternity wards in the city of Campinas in the State of São Paulo, the prevalence of a deficient serum retinol concentration (<0.70 µmol/L) was 14.6% (15), and in a teaching hospital in the city of Ribeirão Preto, 25% of neonates were reported to be deficient (26).

Although cutoffs across studies differ with respect to reporting inadequacy or deficiency, these studies, including ours, appear to reveal a prevalence of vitamin A deficiency of 15-25% among infants born in hospital facilities across geographic regions and socioeconomic levels in Brazil.

In this study, the maternal serum vitamin A levels explained a small percentage—7% of variation in newborns. This modest association could be reflecting homeostatic regulation of vitamin A transfer between the mother and the foetus. Another possible explanation according to Rondó *et al*. (15) is that the correlation of retinol concentration between maternal cord and maternal blood sources occur only when maternal vitamin A deficiency is moderate. In our series, the retinol concentration levels remained within a marginal range. Nonetheless, inadequate maternal consumption of vitamin A and low serum retinol concentrations during gestation appear to influence vitamin A status of the neonate (20) and composition of breastmilk (27). Therefore, women with a chronically low intake of vitamin A may be expected to have infants with low liver reserves of this vitamin, and possibly a higher risk of being vitamin A-deficient in the first months of life (17).

Neonatal VAD has been linked to low birthweight, although a previous review of this relationship has suggested that it was not likely to be causal (28). An association with serum retinol was not observed in our study population for either weight or length.

This finding may have been influenced by the gestational age at birth as all children were born at full term. However, Rondó *et al.* (2) reported that smaller neonates have lower concentrations of serum retinol than larger neonates.

The lack of association between neonatal vitamin A status and income, maternal education and home sanitation, can possibly be explained by a greater importance of materno-foetal exchange biology that is not susceptible to socioeconomic influences.

A greater number of pregnancies is correlated with greater nutritional depletion in mothers that could compromise the nutritional status of vitamin A. However, it is important to emphasize that these mothers had an opportunity for supplementation in the immediate post-delivery period, in accordance with Brazil's National Vitamin A Supplementation Programme, which has expanded significantly in the context of this study.

The negative association between the concentration of retinol in the umbilical cord and concentration of haemoglobin was an unexpected result. There seems to be a synergic effect between vitamin A and iron, in which vitamin A may enhance the mobilization of iron, thereby favouring haemoglobinogenesis (29,30). This synergic effect has been observed in controlled clinical trials (31-33).

Vitamin A deficiency occurs in 15-25% of newborns delivered at term in maternity hospitals in Brazil, signalling a need for greater attention to this potential nutritional problem. While the health implications of this level of deficiency remain unclear, our findings emphasize the potential importance of dietary and supplemental strategies for preventing this deficiency in pregnant women and young infants. Encouraging breastfeeding in the first six months of life and promoting adequate intakes of foods rich in vitamin A during pregnancy and lactation are essential steps in addressing this nutritional deficiency.

## ACKNOWLEDGEMENTS

The authors gratefully acknowledge the Ministries of Health (Convention no. 4807/2005) and Science and Technology (MCT) (Convention no. 01.0265.00/2005) of Brazil for financial support to this research as well as the Centre for Research on Micronutrients (CIMICRON) and the Instituto de Medicina Integral Professor Fernando Figueira for logistical support.

## References

[1] Wolf G (2001). The Discovery of the visual function of vitamin A. J Nutr.

[2] Rondó PHC, Abbott R, Tomkins AM (2001). Vitamin A and neonatal anthropometry. J Trop Pediatr.

[3] Semba RD (1994). Vitamin A, immunity, and infection. Clin Infect Dis.

[4] Klemm RDW, Labrique AB, Christian P, Rashid M, Shamim AA, Katz J (2008). Newborn vitamin A supplementation reduced infant mortality in rural Bangladesh. Pediatrics.

[5] Beaton GH, Martorell R, Aronson KA, Edmonston B, McCabe G, Ross A (1994). La suplementación con vitamina A y la morbilidad y mortalidad infantil en los países en desarrollo1. Bol Oficina Sanit Panam.

[6] West KP (2003). Vitamin A deficiency disorders in children and women. Food Nutr Bull.

[7] World Health Organization (2009). Global prevalence of vitamin A deficiency in populations at risk 1995-2005: WHO global database on vitamin A deficiency.

[8] da Silva Diniz A, Santos LMP (2000). Hipovitaminose A e xeroftalmia. J Pediatr.

[9] Saunders C, Ramalho RA, de Lima APPT, Gomes MM, Campos LF, dos Santos Silva BA (2005). Association between gestational night blindness and serum retinol in mother/newborn pairs in the city of Rio de Janeiro, Brazil. Nutrition.

[10] Biswas AB, Mitra NK, Chakraborty I, Basu S, Kumar S (2000). Evaluation of vitamin A status during pregnancy. J Indian Med Assoc.

[11] Radhika MS, Bhaskaram P, Balakrishna N, Ramalakshmi BA, Devi S, Kumar BS (2002). Effects of vitamin A deficiency during pregnancy on maternal and child health. BJOG.

[12] Christian P, West KP, Khatry SK, LeClerq SC, Kimbrough-Pradhan E, Katz J (2001). Maternal night blindness increases risk of mortality in the first 6 months of life among infants in Nepal. J Nutr.

[13] Christian P1, West KP, Khatry SK, Kimbrough-Pradhan E, LeClerq SC, Katz J (2000). Night blindness during pregnancy and subsequent mortality among women in Nepal: effects of vitamin a and β-carotene supplementation. Am J Epidemiol.

[14] Christian P, West KP, Khatry SK, Katz J, Shrestha SR, Pradhan E (1998). Night blindness of pregnancy in rural Nepal—nutritional and health risks. Int J Epidemiol.

[15] Rondo PH, Abbott R, Rodrigues LC, Tomkins AM (1995). Vitamin A, folate, and iron concentrations in cord and maternal blood of intra-uterine growth retarded and appropriate birth weight babies. Eur J Clin Nutr.

[16] Dimenstein R, Trugo NM, Donangelo CM, Trugo LC, Anastácio AS (1996). Effect of subadequate maternal vitamin-A status on placental transfer of retinol and beta-carotene to the human fetus. Biol Neonate.

[17] Sivaprasadarao A, Findlay JBC (1988). The interaction of retinol-binding protein with its plasma-membrane receptor. Biochem J.

[18] Dancis J, Levitz M, Katz J, Wilson D, Blaner WS, Piantedosi R (1992). Transfer and metabolism of retinol by the perfused human placenta. Pediatr Res.

[19] Stoltzfus RJ, Underwood BA (1995). Breast-milk vitamin A as an indicator of the vitamin A status of women and infants. Bull World Health Organ.

[20] Underwood BA (1994). Maternal vitamin A status and its importance in infancy and early childhood. Am J Clin Nutr.

[21] Furr HC, Tanumihardjo SA, Olson JA (1992). Training manual for assessing vitamin A status by use of the modified relative dose response and the relative dose response assays.

[22] World Health Organization (1995). Using immunization contacts as the gateway to eliminating vitamin A deficiency: a policy document Rev. ed.

[23] World Health Organization (2001). Iron deficiency anaemia: assessment, prevention and control. A guide for programme managers.

[24] Szarfarc SC (1974). Anemia ferropriva em parturientes e recém-nascidos. Rev Saúde Públ.

[25] Puffer RR, Serrano CV (1987). Patterns of birth weight.

[26] El Beitune P, Duarte G, Vannucchi H, Quintana SM, Figueiró-Filho EA, de Morais EN (2004). [Serum vitamin A during pregnancy and effects on obstetrics and perinatal outcomes in HIV infected pregnant women]. Arch Latinoam Nutr.

[27] Ortega RM, Andrés P, Martínez RM, López-Sobaler AM (1997). Vitamin A status during the third trimester of pregnancy in Spanish women: influence on oncentrations of vitamin A in breast milk. Am J Clin Nutr.

[28] West KP Public health impact of preventing vitamin A deficiency in the first six months of life. Delange FM West KP Jr. editors Micronutrient deficiencies in the first months of life.

[29] Bloem MW (1995). Interdependence of vitamin A and iron: an important association for programmes of anaemia control. Proc Nutr Soc.

[30] Jiang S, Wang C-x, Lan L, Zhao D (2012). Vitamin A deﬁciency aggravates iron deﬁciency by upregulating the expression of iron regulatory protein-2. Nutrition.

[31] Suharno D, West CE, Muhilal, Karyadi D, Hautvast JG (1993). Supplementation with vitamin A and iron for nutritional anaemia in pregnant women in West Java, Indonesia. Lancet.

[32] Kolsteren P, Rahman SR, Hilderbrand K, Diniz A (1999). Treatment for iron deficiency anaemia with a combined supplementation of iron, vitamin A and zinc in women of Dinajpur, Bangladesh. Eur J Clin Nutr.

[33] Semba RD, Bloem MW (2002). The anemia of vitamin A deficiency: epidemiology and pathogenesis. Eur J Clin Nutr.

